# Endoscopic endonasal resection of an epidermoid cyst in the cavernous sinus: A case report and literature review

**DOI:** 10.3389/fonc.2022.972573

**Published:** 2022-12-23

**Authors:** Yinzi Wu, Zhimin Li, Jun Gao, Yong Yao, Renzhi Wang, Xinjie Bao

**Affiliations:** Department of Neurosurgery, Peking Union Medical College Hospital, Chinese Academy of Medical Sciences and Peking Union Medical College, Beijing, China

**Keywords:** parasellar lesion, cavernous sinus (CS), epidermoid cyst, endoscopic surgery, endoscopic endonasal transcavernous approach

## Abstract

**Background:**

Epidermoid cysts of cavernous sinus (CS) are rare congenital neoplasms of the central nervous system. In previous literature reports, the treatment for CS epidermoid cysts was mainly microsurgical resection, and the surgical methods included simple microsurgery and endoscope-assisted microsurgery. The present case report demonstrates the first case of complete resection of a CS epidermoid cyst by a simple endoscopic endonasal transcavernous (EET) approach.

**Case presentation:**

A 54-year-old woman presented with chronic persistent headaches and occasional syncope. Brain MRI demonstrated a space-occupying lesion of the left CS, and digital substruction angiography (DSA) showed a small aneurysm at the beginning of the left ophthalmic artery. Thrombotic therapy of carotid–ophthalmic aneurysms was performed first, and the patient underwent resection of the CS lesion secondary. Considering the location of the lesion and the neuroendoscopy technology and experience of the doctor, we made bold innovations and used an EET approach to achieve complete resection of the lesion. The postoperative pathological results were consistent with the characteristics of epidermoid cyst. During the 1-year follow up, the patient showed no apparent signs of recurrence on head MRI.

**Conclusion:**

Epidermoid cyst of cavernous sinus is a rare benign occupying lesion in cavernous sinus. Reviewing the previous literature, the main treatment is microneurosurgery, and neuroendoscopy is only used as an auxiliary equipment. We present the first case of complete endoscopic resection of CS epidermoid cyst by EET approach according to CARE guidelines, aiming to share the new surgical plan for CS epidermoid cyst and provide more surgical options for this disease for neurosurgery colleagues.

## Background

Intracranial epidermoid cysts, as slow-growing congenital benign central nervous system tumors, are often asymptomatic in the early stage and are found because of intracranial mass effects, cranial nerve injury, or epileptic seizures (1). The best treatment option for intracranial epidermoid cysts is surgical resection, including excision of the cyst contents and total or subtotal capsule resection (2). By reviewing the past literature, we found that the published case reports on CS epidermoid cysts all had surgical methods involving microsurgery through various approaches, either alone or assisted by an endoscope (3–7). Here, we report a case in which complete resection of an epidermoid cyst in the CS was achieved by a simple endoscopic endonasal transcavernous (EET) approach.

## Case presentation

**Figure 1:**
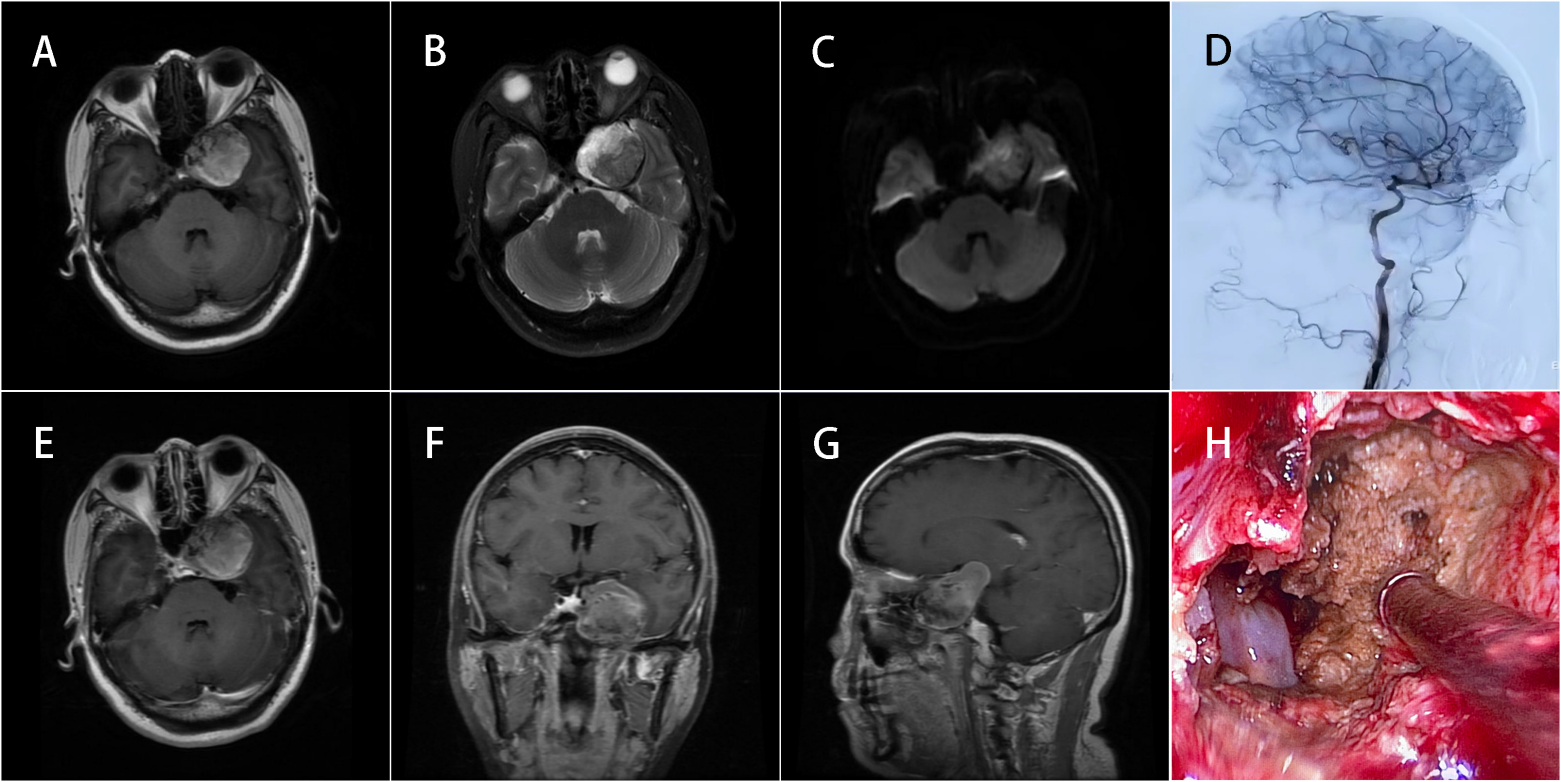
MRI of the brain with and without contrast. **(A)** The left CS lesion was approximately 34 mm × 30 mm in size with a clear boundary but showed mixed signals that were mostly hyperintense on T1-weighted imaging. **(B)** Hypointense signals can be seen on T2-weighted imaging. **(C)** Heterogeneous signals appeared on DWI. **(E–G)** No obvious gadolinium enhancement was noted. **(D)** DSA confirmed that the embolization effect on the aneurysm was satisfactory and the shape of other intracranial vessels was smooth. **(H)** Endoscopic findings revealed that the lesion contents comprised tan mushy substances.

A 54-year-old woman reported a long-term history of intermittent pain in the bilateral temples with no other symptoms but once syncope. Physical and neurologic examination showed no abnormalities. Brain MRI with contrast revealed a 32 mm × 27 mm left CS mass with a clear boundary but mixed signals. Digital substruction angiography (DSA) images indicated smooth running of the internal carotid artery (ICA) but an aneurysm of approximately 4 mm × 2.5 mm at the origin of the left ophthalmic artery. After evaluation, we developed a step-by-step treatment plan: the patient would undergo embolization of the aneurysm first, followed by resection of the left CS lesion. Two years after the aneurysm embolization, brain MRI demonstrated that the lesion had increased (**Figure 1**).

## Treatment and outcome

The patient underwent EET surgery. After removing the left middle nasal concha, ethmoid vesicle, and uncinate process, wide lateral sphenoidotomies were performed to expose the left anterior inferior wall of the CS. The bone overlying the left anterior wall of the CS was removed, and the lesion had partially protruded into the sphenoid sinus. The capsule of the lesion was cut open and a large amount of brown paste including a few yellowish-tan liquids spilled over, which was different from the white waxy content of the classic epidermoid cysts. It may be fresh and old hemorrhage. The different composition of the cystic contents was related to the variation in radiological features of the epidermoid cysts, which may make preliminary diagnosis difficult.

**Figure 2:**
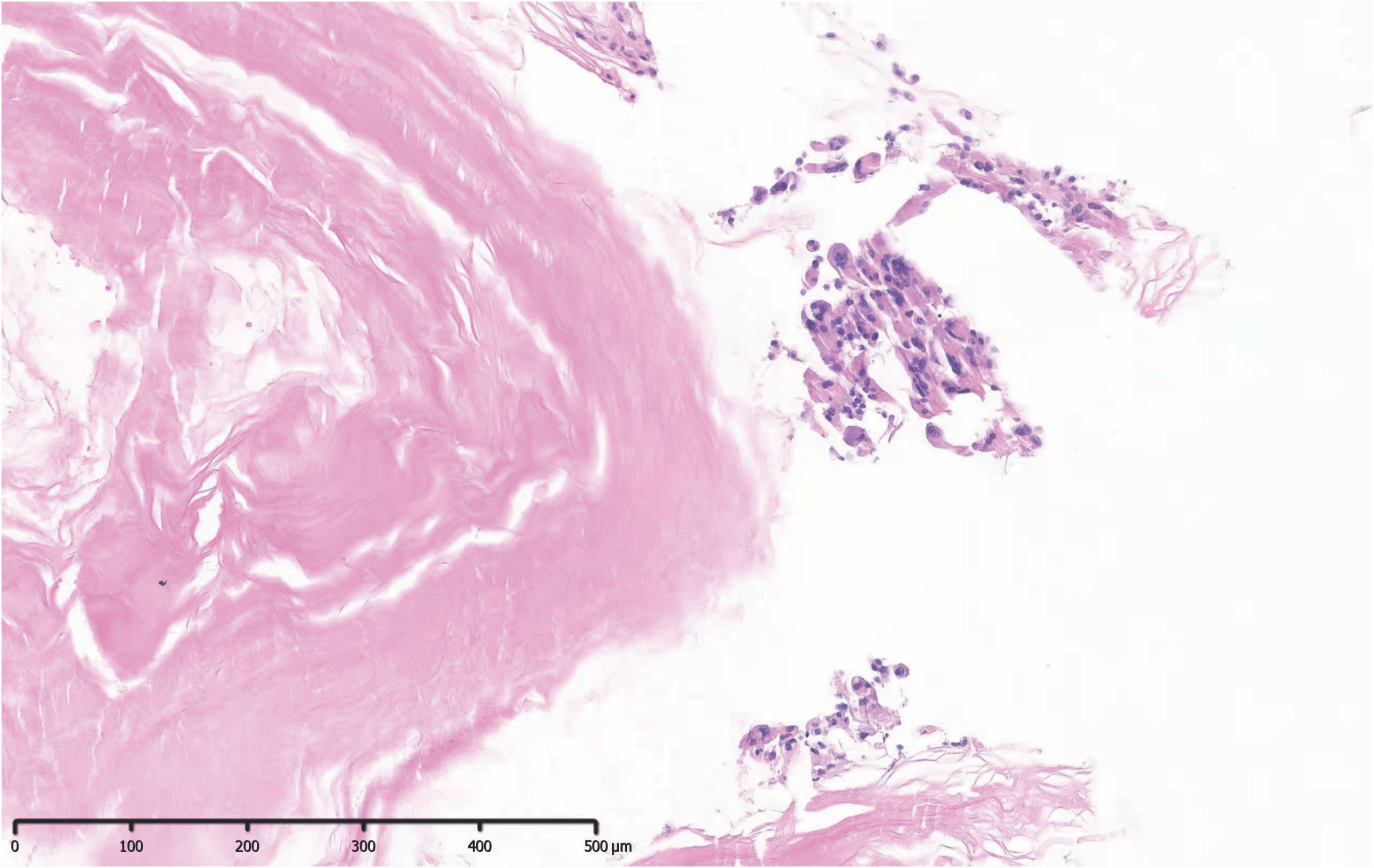
Pathological examination revealed keratinoid and epidermoid cells, which were consistent with the pathological features of an epidermoid cyst.

During the procedure, we used 0°C and 30°C endoscopes with different angles to expose the CS without any blind area and maximize the lesion resection. The lesion was located in the interdural space of the lateral wall of the CS. After the lesion contents were completely resected, we found that the left internal carotid artery (ICA) was inside the lesion and the III, IV, and V1 nerves were on the outside of the lesion. To protect the nerves and ICA, subtotal capsule resection was achieved. At the end of the operation, the iodoform gauze was filled in the lesion cavity, which can not only have the effect of bacteriostasis, but also form a sinus gradually between the lesion cavity and the nasal cavity to minimize the possibility of recurrence. The contents and capsule of the lesion were sent for pathology examination, and the result was consistent with an epidermoid cyst (**Figure 2**).

**Figure 3:**
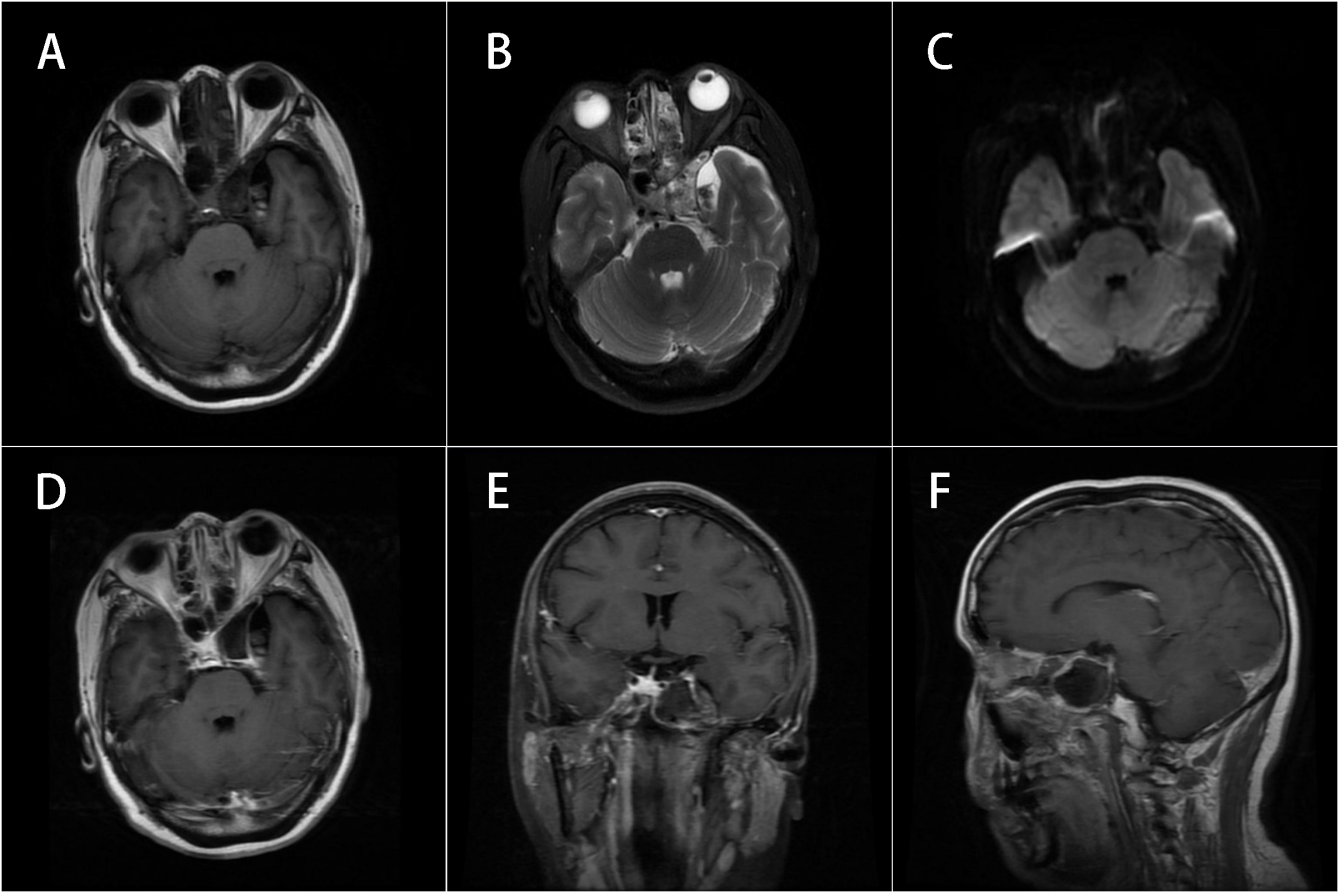
MRI of the brain with and without contrast. **(A–C)** Corresponded to **Figure 1A–C** and **(D–F)** corresponded to **Figure 1E–G** respectively. The lesion had been completely resected and the abnormal signals observed on the preoperative imaging had disappeared.

No new neurological deficits were observed after operation, and no cerebrospinal fluid leakage or central nervous system infection occurred. After the operation, the patient’s headache almost disappeared. We thought that it was attributed to the disappearance of the space-occupying effect caused by this massive epidermoid cyst and the relief of the compression on the surrounding tissue, including the brainstem and the nerves in the cavernous sinus. Through the screenage shown by the intraoperative video and the comparison of preoperative and postoperative MRI images, the effect of surgical resection is noticeable. Postoperative head MRI confirmed that the lesions were completely resected (**Figure 3**). The patient was discharged on day 3 after the operation. Only mild nasal discomfort after the operation, short hospital stay, and total resection suggested by imaging re-examination made patients very satisfied with this diagnosis and treatment. During the 1-year follow up, the patient recovered well and had no signs of recurrence.

## Discussion and conclusion

Epidermoid cysts, also known as primary cholesteatomas (8), are currently thought to arise from ectodermal remnants that fail to degenerate after neuroembryonic development in the fifth week of gestation (2). Intracranial epidermoid cysts are uncommon, accounting for approximately 0.2%–1.8% of intracranial tumors and are usually located in the cerebellopontine angle, prepontine cistern, and middle cranial fossa (7, 9). Epidermoid cysts originating in the CS are even more rare, accounting for only 2% of intracranial epidermoid cysts (5). The growth of epidermoid cysts occurs through accumulation of keratin and cholesterol from peeling of the lining epithelium, which can wrap or compress the nearby cranial nerves and vascular system (1). As the cysts grow slowly, their clinical symptoms often appear later in life. Frequently, the cysts are finally discovered through intracranial mass effects, aseptic encephalitis aroused by their rupture (4, 10, 11), or head examinations for other diseases.

MRI is the examination of choice for diagnosis of epidermoid cysts (11). Typical epidermoid cysts show hypointensity on T1-weighted imaging and hyperintensity on T2-weighted imaging, and the opposite findings are atypical. In a statistical analysis of 428 patients with intracranial epidermoid cysts in their hospital in 2012, Ren et al. found that atypical epidermoid cysts were usually larger and more prone to spontaneous bleeding than typical epidermoid cysts (12). According to Tsurushima et al., lipids and methemoglobin can cause hypointensity on T1-weighted imaging, while accumulation of hemosiderin can cause hypointensity on T2-weighted imaging, leading to atypical MRI findings (13). The lesion in our patient showed mixed signals on MRI, which is atypical and made the preoperative diagnosis not precise. We considered the mass possibly being a giant aneurysm with thrombosis. We performed DSA, indicating smooth intracranial vessels’ smooth running. Vascular disease was ruled out. The patient had no obvious clinical symptoms. Therefore, the texture of this lesion might be soft. Even though its volume is not small, it did not produce an apparent occupying effect. We still considered it more likely to be an epidermoid cyst consequently. The lesion in our patient showed mixed signals on MRI, which is atypical and made the preoperative diagnosis not precise. Nevertheless, we confirmed that the lesion was an epidermoid cyst during surgery. Intraoperative aspiration of the lesion contents showed tan components, which was different from the white pearly contents of typical epidermoid cysts, indicating possible signs of bleeding.

Surgery is the best treatment option for CS epidermoid cysts (7). We performed a systematic review of related literature using PubMed in accordance with the Preferred Reporting Items for Systematic Reviews and Meta-Analyses (PRISMA) guidelines. Various combinations of the following terms were used to search the literature: epidermoid cyst, cavernous sinus, intracranial, central nervous system, endoscopy, endoscopic, endonasal, and transsphenoidal. Our search strategy initially identified 62 articles. After screening titles and abstracts, a second filter of the collected literature according to the tumor location and surgical method was performed. Finally, it included 13 English literature, a total of 48 cases of intracranial cavernous sinus epidermoid cyst. We summarized the clinical symptoms, surgical approach, operation time, degree of resection, postoperative complications, and long-term prognosis of these cases (**Table 1**). Through a search of previous literature, we found that 48 cases were reported in detail. All these patients were treated with microneurosurgery, including simple microsurgery and endoscopic-assisted surgery (**Table 1**), and total or subtotal resection was achieved. In four patients, the part of lesions were invisible under simple microsurgery but were completely resected with the aid of an endoscope (5, 6). In a report on patients with CS epidermoid cysts, data analyses showed that 7.9% of the patients developed new oculomotor paralysis after microsurgery, while 22.6% developed aseptic meningitis or septic meningitis (5). Although these postoperative complications were all resolved and the patients were finally discharged, the complications prolonged the patients’ length of hospital stay.

**Table 1:** Case summaries for 49 patients with CS epidermoid cysts.

Authors (Year)	NC	Symptoms	Approach	MOT	Surgical removal	Postop outcome	Complication	PHS	Long-term outcome	FU	Recurrence
Kline & Galbraith, 1981 (14)	1	Headache, diplopia, CN III and V1 palsy	Frontotemporal craniotomy	>4 h	Subtotal	Right periorbital pain completely resolved	Complete right III CN palsy	>7 days	CN III palsy recovered, CN V1 dysfunction resist	6 months	No
Ikezaki et al., 1992 (15)	1	Left CN VI palsy	Pterional approach	>4 h	Subtotal	–	No	14 days	CN VI palsy partially recovered	4 months	No
Tatagiba et al., 2000 (11)	1	Right ocular palsy, periorbital pain, pain in the right CN V1 branch, hypesthesia of the CN V2	Frontotemporal craniotomy	>4 h	Subtotal	Pain completely resolved, mode-rate hypesthesia of CN V2	–	7 days	CN III palsy disappeared completely	5 months	No
Gharabaghi et al., 2005 (4)	1	Chronic headache, hypesthesia of the right CN V1 and V2	Frontotemporal craniotomy	>4 h	Total	–	No	5 days	–	1 year	No
Bonde & Goel, 2008 (3)	1	Trigeminal neuralgic pain, mild hypoesthesia of the left CN V1 and V2	Basal anterior subtemporal extradural approach	>4 h	Total	Pain completely resolved	–	>7 days	CN V palsy recovered partially	3 years	–
Ghaemi et al., 2010 (16)	1	Double vision, CN III palsy	Pterional approach	>4 h	Subtotal	Improvement of CN III palsy	No	>7 days	–	1.5 years	Yes
Chung et al., 2012 (6)	1	Headache, diplopia, visual disturbance, CN III palsy	Endoscope-assisted right frontotemporal craniotomy	>4 h	Total	Pain completely resolved	–	>7 days	CN III dysfunction resist	2 years	No
Ren et al., 2012 (12)	1	Dizziness, right eye discomfort, right face numbness	Frontotemporal craniotomy	>4 h	Subtotal	–	No	14 days	–	–	–
Kuroi et al., 2014 (17)	1	CN VI palsy	Subtemporal approach	>4 h	Total	–	–	>7 days	CN VI palsy partially recovered	–	No
El-Kalliny et al., 1992 (18)	2	Diplopia, CN V1 palsy	1st op: frontotemporal craniotomy	>4 h	Subtotal	Diplopia resolved in the primary gaze but persisted in the upward gaze	–	>7 days	Progressive CN III palsy	2 years	Yes
			2nd op: frontotemporal craniotomy	>4 h	Subtotal	Diplopia in primary gaze again resolved while diplopia in upward gaze remained unchanged	–	>7 days	–	–	–
		Headaches, diplopia, visual disturbance, pain of CN V2, CN III and IV palsy	Frontotemporal craniotomy	>4 h	Subtotal	Improvement in visual acuity	–	>7 days	–	1 year	Yes
Pamir et al., 2006 (19)	2	–	Pterional approach	>4 h	Total	Improvement of CN function	NO	>7 days	–	1 year	No
Wang et al., 2013 (9)	4	Vision blurred, hypesthesia of the left CN V1 and V2	Frontotemporal craniotomy	>4 h	Total	Improvement in all symptoms	–	>7 days	CN V palsy resist	7 years	No
		Headache, right CN V palsy			Total	Pain relieved, improvement of CN V palsy	–	>7 days	CN V palsy recovered partially	4 years	No
		Headache, left CN III and V1 and V2 palsy			Subtotal	Pain relieved, improvement of CN III and V1 and V2 palsy	–	>7 days	–	3 years	No
		Headache, vision blurred, left CN IV and V palsy			Total	Improvement of CN IV and V palsy	–	>7 days	–	5 years	No
Zhou et al., 2018 (5)	31	Facial numbness or hypesthesia, absent corneal reflex, diplopia, temporal muscle atrophy, CN III and VI deficit, trigeminal neuralgia	Extradural only Intradural only Combined	>4 h	Total (16) Subtotal (15)	Symptoms remained similar (74.2%) Improvement of CN function (16.1%)	Aseptic meningitis (16.1%) Septic meningitis (6.5%) CN III palsy (9.7%)	>7 days	Diplopia remission or CN III palsy recovered (36%)	4.6± 3.0 years	No
This study	1	Intermittent pain in bilateral temples	Endoscopic endonasal transcavernous approach	3 h	Total	Pain completely resolved	No	3 days	No special	1 year	No

NC, number of cases; MOT, mean operative time; PHS, postoperative hospital stay; FU, follow-up; -, not mentioned.

Compared with traditional microsurgery, the EET approach, as an alternative to the medial CS approach (20), dramatically reduces the surgical trauma for patients, thereby markedly reducing the risk of postoperative intracranial infection. With the continuous improvement of endoscopic technology and equipment, experienced neurosurgeons use different endoscopes to achieve more extensive visualization of the surgical field than microscopes, so that the total resection rate of tumor under endoscopy has been further improved, and even avoid the possibility of tumor remnant caused by blind area under the microscope (21). In this case, we used a 0°C and a 30°C endoscope to inspect every corner of the lesion and achieved total excision of the contents and subtotal resection of the capsule. At the same time, the excellent visualization also significantly reduces the probability of vascular and nerve injury (5–7, 22). Compared with craniotomy microsurgery, we skillfully used the EET approach to create the drainage sinus of the lesion cavity, which reduced the recurrence probability of the lesion. Furthermore, patients have less postoperative discomfort and shorter recovery time, meaning that their medical experience is greatly improved.

Simple endoscopic resection of CS epidermoid cysts has apparent advantages, but also limitations. First, it may be more suitable for the treatment of those with less ICA infiltration (20). Owing to the small available operative area, it is much challenging to control bleeding due to ICA injury compared with microsurgery (23). Thus, it puts forward higher requirements for the endoscopic technology and experience of neurosurgeons. Second, some researchers mentioned that the heat generated by the light source of the endoscope could cause irreversible thermal damage to the surrounding cranial nerves (6). Additionally, because there is no published similar case of endoscopic endonasal resection of the CS epidermoid cysts for reference, long-term follow-up of this patient and more similar cases are needed to improve the statistical analysis of the long-term outcome of this surgical treatment of the CS epidermoid cysts.

In summary, because the contents of epidermoid cysts are soft wax-like substances, they are suitable for excision using the suction function of an endoscope, and endoscopic surgery has apparent advantages such as less trauma and quicker recovery. With the continuous advancement in endoscopic surgery types and neurosurgeon mastery of endoscopic technology, minimally invasive endoscopic surgery may become an ideal choice for CS epidermoid cysts in the future (5).

## Data availability statement

The original contributions presented in the study are included in the article/**Supplementary Material**. Further inquiries can be directed to the corresponding author.

## Ethics statement

The studies involving human participants were reviewed and approved by the Ethics Committee of PUMCH. The patients/participants provided their written informed consent to participate in this study. Written informed consent was obtained from the individual(s) for the publication of any potentially identifiable images or data included in this article.

## Author contributions

YW wrote the first draft of the manuscript. XB wrote sections of the manuscript. All authors contributed to manuscript revision, read, and approved the submitted version.
